# Raspberry Pi Platform Wireless Sensor Node for Low-Frequency Impedance Responses of PZT Interface

**DOI:** 10.3390/s22249592

**Published:** 2022-12-07

**Authors:** Quang-Quang Pham, Quoc-Bao Ta, Jae-Hyung Park, Jeong-Tae Kim

**Affiliations:** 1 Department of Ocean Engineering, Pukyong National University, 45 Yongso-ro, Nam-gu, Busan 48513, Republic of Korea; 2 CNS Solution Co., Ltd., 21 Century City Office, 312 Suyeong-ro, Nam-gu, Busan 48513, Republic of Korea

**Keywords:** Raspberry Pi, wireless sensor node, impedance response, PZT interface, stress variation

## Abstract

A wireless impedance monitoring system, called SSeL-Pi, is designed to have cheap, mobile, and handy practical features as compared to wired commercial impedance analyzers. A Raspberry Pi platform impedance sensor node is designed to measure signals at a low-frequency range of up to 100 kHz. The low-frequency impedance measurement via the proposed node has been combined with a new PZT interface technique for measuring local responses sensitive to structural damage. The new PZT interface can work as a surface-mounted or embedded sensor, and its local dynamic characteristics are numerically analyzed to pre-determine an effective impedance resonant frequency range of less than 100 kHz. Next, a software scheme was designed to visualize the input/output parameters of the proposed SSeL-Pi system (i.e., Raspberry Pi platform and PZT interface) and automate signal acquisition procedures of the impedance sensor node. The calibration for impedance signals obtained from the proposed system was performed by a series of procedures, from acquiring real and imaginary impedance to adjusting them with respect to a commercial impedance analyzer (HIOKI-3532). The feasibility of the wireless impedance monitoring system was experimentally evaluated for PZT interfaces that were subjected to various compressive loadings. The consistent results analyzed from signals measured by the SSeL-Pi and HIOKI 3532 systems were observed. Additionally, the strong relationships between impedance features (frequency shift and RMSD index) and compressive stresses of the PZT interfaces showed the potential for axial force/stress variation monitoring in real structures using the Raspberry Pi platform impedance sensor node and developed PZT interface.

## 1. Introduction

Electromechanical impedance (EMI)-based techniques have been developed for structural health monitoring (SHM) in civil, mechanical, and aerospace engineering areas [1,2,3]. The fundamental theory of the EMI-based methods was developed by Liang et al. [4], and it was successively followed by many researchers [5,6]. However, the field implementation of the techniques is limited due to the lack of affordable hardware. As they are designed for the laboratory environment, most impedance analyzers (e.g., HIOKI 3532 or HP4149A) are bulky and expensive but unsuitable for use in the field. Therefore, there is a need to develop portable, handy, and inexpensive measurement units for impedance-based SHM of real structures in the field.

Wireless impedance monitoring methods have been developed for SHM [7,8,9,10,11,12,13,14,15]. Mascarenas et al. [7] proposed a prototype of a wireless impedance sensor node for structural health monitoring. The developed sensor node used an AD5933 impedance chip [16] for measuring the impedance signals of a piezoelectric (PZT) transducer, an ATmega128L microcontroller for computing, and a 2.4 GHz Xbee radio-frequency telemetry module for wireless communication. Many researchers have then followed and improved the initial prototype to obtain a low-cost multi-functional wireless impedance sensor node. Park S. et al. [10] improved the prototype by adding some functions such as a memory card slot, multi-channel measurement, and temperature recording. Min et al. [11] embedded the algorithms on the microcontroller for structural damage detection/sensor self-diagnosis. Kim et al. [13] developed an SSeL-I impedance sensor board operated on an Imote2 platform to enhance the wireless monitoring capacity. As a high-performance sensor platform, the Imote2 [17] consists of a low-power microcontroller of 416 MHz clock speed, 32 MB SDRAM (synchronous dynamic random-access memory), and a radio transceiver of 250 kb/s transmission rate with 16 channels in the 2.4 GHz band. The Imote2 platform impedance sensing system (called Imote2/SSeL-I) was applied for local damage detection in structural connections [13,15,18] and prestress-loss monitoring in tendon anchorage [19,20]. 

Recently, Raspberry Pi has been developed for a commercial microcomputer [21,22]. As compared to the Imote2 platform, Raspberry Pi has many advanced features, such as a powerful microprocessor of 1.5 GHz clock speed, 1~8 GB SDRAM, and a wireless LAN with 600 Mb/s transmission rate in the 2.4/5.0 GHz bands. The Raspberry platform simultaneously supports multiple sensors via a 40-pin GPIO (general-purpose input/output) header. For example, a unit of Raspberry Pi costs about US$80 for a model with 4 GB SDRAM. As a cheap, powerful, and multi-functional unit, Raspberry Pi is an effective platform for controlling distributed sensors to continuously monitor structural responses [23,24,25,26]. Mahmud et al. [24] used a Raspberry Pi to generate an excitation signal on a PZT sensor, detect damage in a metal plate, and send a structural health status to an Internet server. This study used a combination of pitch-catch and pulse-echo techniques for monitoring the structure’s health. Ghosh et al. [25] employed a Raspberry Pi as a computing unit to code the piezoelectric sensors and receive the data from these sensors in structural health monitoring for concrete beams. Meng and Zhu [26] combined a Raspberry Pi 4 and a microelectromechanical systems accelerometer to form an Internet of Things sensing system for vibration monitoring.

So far, the Rasberry Pi platform has not been used for wireless impedance-based SHM. At least three techniques should be developed to make it feasible. Firstly, a data acquisition system should be integrated with a miniature impedance analyzer. A sensing module ‘PmoldIA’ for measuring impedance signals up to 100 kHz [27] (which costs about US$50) is a good choice for the Raspberry Pi platform. Secondly, the operating software of the Raspberry Pi platform should be programmed for autonomous measurement and feature extraction. Thirdly, the data acquisition protocol in the impedance sensor node should be calibrated based on the commercial impedance analyzer.

In this study, a new wireless impedance monitoring system, called SSeL-Pi, is designed to have cheap, mobile, and handy practical features as compared to wired commercial impedance analyzers (e.g., HIOKI 3532 or HP4149A). A Raspberry Pi platform sensor node is designed to acquire impedance signals at a low-frequency range of up to 100 kHz. The low-frequency impedance measurement was combined with a new PZT interface technique, which was developed following the study of Huynh et al. [28]. The new PZT interface helps pre-determine effective frequency ranges of less than 100 kHz, which is used for measuring local responses sensitive to structural damage. A software scheme was designed to operate the proposed SSeL-Pi system (i.e., Raspberry Pi platform and PZT interface). Next, a calibration procedure was designed for the impedance sensor node. The calibration results from the proposed system were compared to signals obtained from the commercial impedance analyzer system (HIOKI 3532). Then, the feasibility of the newly designed impedance sensing system was experimentally evaluated for PZT interfaces subjected to various compressive loadings. 

This paper is organized as follows: (i) Section 2 presents the development of the hardware, the software, and the PZT interface technique for the proposed SSeL-Pi system; (ii) Section 3 presents the calibration procedures for the monitoring system; (iii) Section 4 presents the experiment performed to evaluate the feasibility of the SSeL-Pi system; and (iv) Section 5 draws the findings and conclusions. The major contributions of this study are described as follows: (i) the newly developed impedance sensor node was developed based on the Raspberry Pi platform; (ii) the new PZT interface model with a low pre-determined frequency range of less than 100 kHz was integrated with the Raspberry Pi platform impedance sensor node; and (iii) the newly developed impedance sensor node was applied to monitor stress variation in concrete structures.

## 2. Design of Raspberry Pi Platform Impedance Monitoring System

According to Nagayama et al. [29], a smart sensor node has five main features: an on-board microprocessor, sensing capability, wireless radio, battery power, and low cost. The sensing capability is implemented by a data acquisition unit that enables a signal generator, amplifier, and anti-aliasing filter. The microcontroller operates on-board computation of signal process and feature extraction for the data acquisition unit. As proposed by Mascarenas et al. [7], the data acquisition procedure for an impedance-based sensor node has three steps. Firstly, an AD5933 impedance chip with a 12-bit analog-to-digital converter (ADC) measures impedance signals from a PZT sensor. Then, impedance signals are stored in data storage (e.g., secure digital card—SD card). And lastly, the stored data are transferred to a master device (e.g., laptop) via the wireless communication module. 

### 2.1. Schematic of Impedance Monitoring System

**Figure 1:**
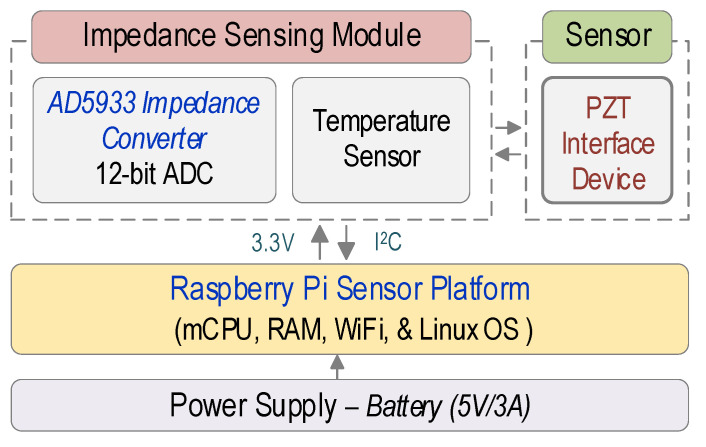
Raspberry Pi platform impedance monitoring system ‘SSeL-Pi’.

In this study, a Raspberry Pi platform impedance monitoring system ‘SSeL-Pi’ was designed as schematized in Figure 1. The main components of the SSeL-Pi system include a power supply, a Raspberry Pi platform [22], an impedance sensing module PmodIA from Digilent [27], and a PZT interface device. The SSeL-Pi system requires a power supply of 5 V/3 A. The Raspberry Pi platform manages a micro-central processing unit (mCPU), a wireless radio (WiFi), and a few other parts described in the following Section 2.2. The Raspberry Pi platform runs on Raspbian, which is an operating software (OS) based on the Debian Linux distribution optimized for the Rasberry Pi family.

The impedance sensing module manages a built-in AD5933 impedance chip with a 12-bit ADC converter. The board is also equipped with a temperature sensor. The built-in AD5933 impedance chip generates frequency-domain signals of less than 100 kHz to the PZT sensor (e.g., PZT 5 A patch). The impedance sensor node was designed to be combined with a new PZT interface device, which was designed following the study of Huynh et al., 2017 [28]. The proposed PZT interface helps ensure impedance signals (less than 100 kHz) that are sensitive to local damage and stress change. The inter-integrated circuit (I^2^C) communication protocol and a power supply (3.3/5 V) are utilized for communicating between the Raspberry Pi platform and the impedance sensing module. The impedance sensing module is connected to the Raspberry Pi platform via GPIO pins [21] (see Figure 1). Additionally, the PZT sensor is connected to the impedance sensing module by electrical wires.

As outlined in Table 1, the specification of the proposed SSeL-Pi system is compared to those of the impedance measurement system HIOKI 3532 [30]. The proposed system can measure an impedance range of up to 10 MΩ with a frequency range of up to 100 kHz [27]. The proposed system is smaller in dimension and weight as compared to the commercial one. The voltage peak-to-peak (V_p-p_) is 2 V_p-p_ for the board PmodIA [27], while it is up to 14 V_p-p_ for HIOKI 3532 [30]. Note that the Raspberry Pi platform sensing system costs around US$280 (in E-commerce, the price for Raspberry Pi 4 B with full options is around US$230 and for PmodIA about US$50), which is much lower than the HIOKI 3532 system. 

### 2.2. Specification of Impedance Monitoring System

#### 2.2.1. Raspberry Pi Platform

A Raspberry Pi is a single-board computer with good features such as small size, low price, Linux operation, and flexible form factor. In this study, a Raspberry Pi 4 B platform was used for the impedance sensing system. Its main specifications are outlined in Table 2. It has a micro central processing unit (mCPU) of 64-bit quad-core Cortex-A72 (1.5 GHz), RAM memory of LPDDR4 4 GB, wireless radio 802.11 ac of WI-FI 2.4/5 GHz, and storage capacity of 16 GB SD card. The Raspberry Pi platform can be powered by a USB-C connection supplying at least 400 mA at 5 V. An on-board 40-pin GPIO header connects externally to the impedance sensing module. Others include USB and HDMI ports for external devices (e.g., keyboard and monitor), CSI and audio ports for video/audio input, and Gigabit Ethernet.

#### 2.2.2. Impedance Sensing Board

**Figure 2:**
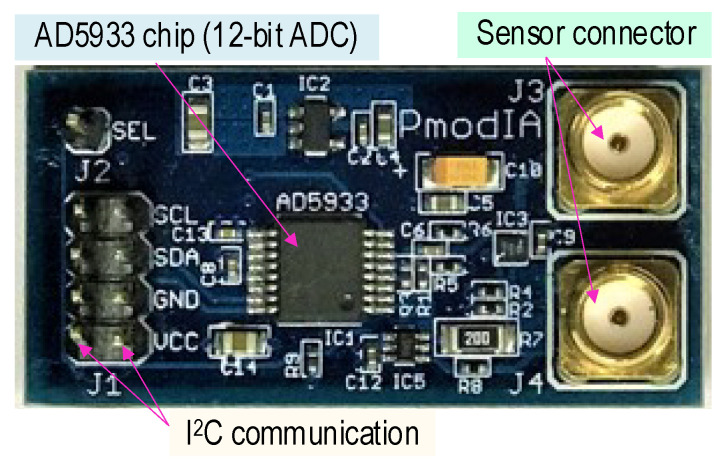
Impedance sensing module ‘PmodIA’.

Figure 2 shows a portable impedance analyzer board (the so-called PmodIA module [27]) which consists of an AD5933 chip, temperature sensor, I^2^C communication ports, and two SMA (subminiature version A) sensor connectors. The Analogue Devices AD5933 chip is a high-precision impedance converter that combines an on-board frequency generator with 12-bit ADC [16,27]. The I^2^C communication has four pins, which are SCL (serial clock), SDA (serial data), GND (ground), and VCC (voltage common collector) (see Figure 2). There is a pair for each type of pin. The impedance measurement using the AD5933 is performed in the following three procedures. Firstly, a frequency generator excites complex dynamic signals at an assigned frequency that is commended externally via the I^2^C communication. Secondly, the response signal is sampled by the on-board ADC. Thirdly, a discrete Fourier transform (DFT) is processed by an on-board DSP (digital signal processor) engine. The DFT algorithm returns a real (R) and imaginary (I) data word at each output frequency. 

As outlined in Table 3, a few parameters of the PmodIA module [27,31,32] were orientated for the measurement of impedance signatures from the PZT interface device (which is described later). The internal oscillator frequency was set at 16.776 mHz to run the device [27]. The current-to-voltage amplifier gain resistor and peak-to-peak voltage were set at 20,000 Ohm and 2 V_p-p_, respectively [31,32]. The programmable gain amplifier (PGA) was ×5 times, and the number of setting time cycles was 100. The supply voltage was 3.3 V. 

#### 2.2.3. Circuit Design of ‘SSeL-Pi’

**Figure 3:**
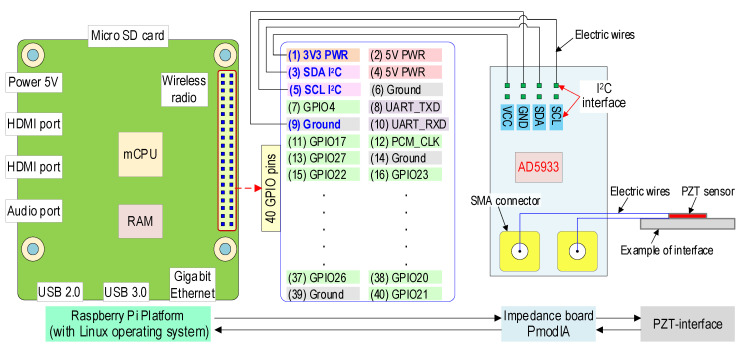
Circuit design of ‘SSeL-Pi’.

Figure 3 shows the connection method between Raspberry Pi 4, the PmodIA, and an example of the PZT interface. First, the PmodIA was linked with Raspberry Pi 4 via I^2^C communication. The SDA and SCL pins of the PmodIA were connected with the GPIO SDA (pin #3) and GPIO SCL (pin #5) of Raspberry Pi 4, respectively. Additionally, the VCC and GND pins of the PmodIA were linked to pin #1 (3V3 PWR) and pin #9 (Ground) of the Raspberry Pi 4, correspondingly. Then, the example of the PZT interface was connected to the PmodIA via the SMA connectors on this board.

#### 2.2.4. PZT Interface for Impedance Measurement

##### Impedance Concept of PZT Interface

Huynh et al. [28] developed a PZT interface technique to monitor the damage in a tendon anchorage induced by the variation of tendon forces. The developed interface, which worked as a beam-like structure, included a flexible part in the middle and two outside fixed parts. The proposed technique helped pre-determine the effective frequency band less than 100 kHz, which was suitable for wireless impedance measurement [7,12]. 

**Figure 4:**
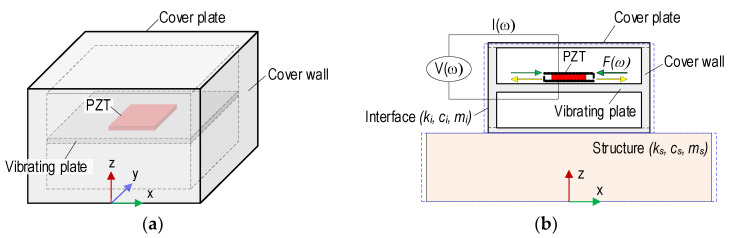

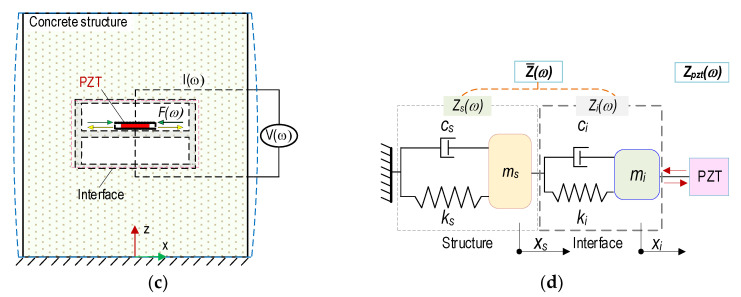
A PZT interface device for impedance monitoring. (**a**) PZT interface, (**b**) Surface-mounted PZT interface on structure, (**c**) Embedded PZT interface in concrete structure, (**d**) 2-DOF impedance model.

By following the above-mentioned study, a new type of aluminum PZT interface was proposed for structural health monitoring using a low pre-determined frequency range (less than 100 kHz) of impedance responses. As shown in Figure 4a, the newly designed PZT interface has a vibrating plate (i.e., flexural section). Then, the fabricated component is covered by two plates on its top and bottom, respectively. The vibrating plate, where the PZT patch is installed, is designed to help the interface fluctuate according to the inverse-piezoelectric behaviors of the PZT patch. With the support and protection of the cover walls and plates (i.e., top and bottom plates), the PZT interface works as a multi-functional sensor: (1) surface-bonded PZT sensor and (2) embedded PZT sensor.

For the surface-bonded sensor (see Figure 4b), the PZT interface could be contacted with a host structure via a bonding layer between the bottom plate of the PZT interface and structural surface. With the protected cover walls and plates, the integrity of the PZT sensor could be ensured during the construction and operation of the host structure. Furthermore, the ambient effects [33,34] on the impedance responses could be reduced.

For the embedded PZT sensor (see Figure 4c), the PZT interface could be embedded in the concrete structures and work as a piezoelectric-based smart aggregate [35]. By embedding into the concrete structures, the PZT interface could directly observe the internal stress variation and concrete transformation induced by the externally applied forces [36]. Furthermore, with the pre-determined frequency range, the impedance measurement using the embedded PZT interface could be processed faster than that using the conventional smart aggregates, which used the trial-and-error method to find the effective monitoring frequency ranges [37]. Thus, the proposed PZT interface could be cost-saving and time-efficient for the impedance monitoring procedure. 

As described in Section 2.2.1 and Section 2.2.2, the Raspberry Pi platform impedance sensor node was designed to measure signals at the frequency range of up to 100 kHz. Thus, the developed measuring system can be combined with the new PZT interface device for low pre-determined frequency impedance monitoring. 

As shown in Figure 4d, the interaction between the PZT interface and target structure could be simplified as a two-DOF impedance model [28]. In the two-DOF impedance model, one DOF stands for the motion of the PZT interface, and the other represents the motion of the inspected structure. The coupled structural-mechanical (SM) impedance $\bar{Z}(\omega )$ of the interface structure at the PZT-driven point can be written as [28]:

(1) $$\bar{Z}(\omega )=\frac{f_{i}(\omega )}{{\dot{x}}_{i}(\omega )}=\frac{K_{11}(\omega )K_{22}(\omega )-K_{12}^{2}(\omega )}{i\omega K_{22}(\omega )}$$

where dynamic stiffness coefficients are derived as $K_{11}=-\omega^{2}m_{i}+i\omega c_{i}+k_{i}$, $K_{12}=-i\omega c_{i}-k_{i}$, and $K_{22}=-\omega^{2}m_{s}+i\omega \left(c_{i}+c_{s}\right)+\left(k_{i}+k_{s}\right)$. The terms m, c, and k are mass, damping coefficient, and spring stiffness.

The subscripts *s* and *i* stand for the inspected structure and interface, respectively. The stiffness coefficients depend on the structural parameters of the inspected structure and interface. The SM impedance of the PZT patch, $Z_{p}\left(\omega \right)$, and that of the interface-inspected structure, $Z_{s}\left(\omega \right)$, are coupled together (Liang et al. 1994 [4]):

(2) $$Z(\omega )=\frac{V}{I}={\left\{i\omega \frac{w_{a}l_{a}}{t_{a}}\left[{\hat{\varepsilon }}_{33}^{T}-\frac{1}{Z_{a}(\omega )/\bar{Z}(\omega )+1}d_{31}^{2}{\hat{Y}}_{11}^{E}\right]\right\}}^{-1}$$

where $w_{a}$, $l_{a}$, $t_{a}$ are geometric constants of the PZT patch; ${\hat{Y}}_{11}^{E}$, $d_{31}$, and ${\hat{\varepsilon }}_{33}^{T}$ are the complex Young’s modulus patch at zero electric fields, the piezoelectric coupling constant, and the complex dielectric constant zero stress, respectively. Once the PZT patch has no changes in mechanical properties or electrical characteristics and no energy loss induced by contact conditions between the interface structure, the SM impedance $Z_{p}\left(\omega \right)$ remains constant. Thus, any changes in the monitored structure (e.g., stress change or structural damage) that cause shifts in impedance frequencies can be measured via the interface technique. 

The 2-DOF impedance model contains resonant peaks in its impedance signatures that represent coupled vibration modes out of the PZT interface structure system. Therefore, the effective frequency bands of the impedance signatures can be predetermined by controlling the structural parameters of the PZT interface. Additionally, the impedance model represents the structural parameters of both the interface device and host structure. Any structural changes (e.g., damage or stress variation) result in a change in the impedance response of the model.

##### Numerical Impedance Response and Local Dynamic Characteristics of PZT Interface

As studied by [28,38], the effective frequency ranges for impedance measurement via the PZT interface have a relationship with the dynamic characteristics of this device. Therefore, the local dynamic characteristics of the PZT interface needed to be examined.

**Figure 5:**
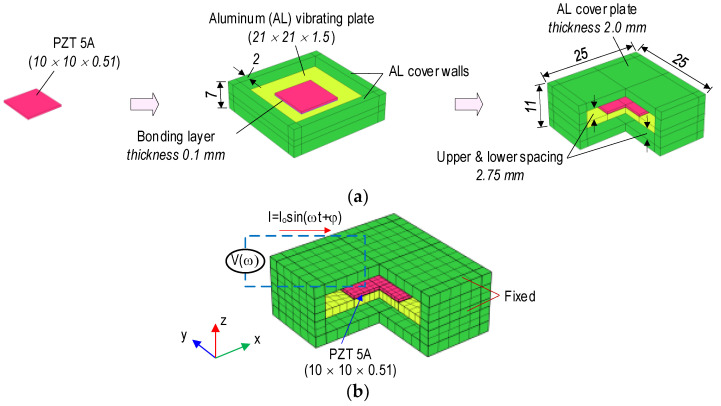
Geometric parameters of the PZT interface (dimension in mm). (**a**) Prototype design, (**b**) Meshing and boundary conditions.

Figure 5a shows a prototype design of the PZT interface. Aluminum was selected for the interface’s material. A PZT 5 A patch, which had a size of 10 × 10 × 0.51 mm, was surface-mounted on the middle of a component including the vibrating plate (21 × 21 × 1.5 mm) and the cover walls (height of 7 mm and thickness of 2 mm). Super glue was used for a bonding layer (0.1 mm thickness). After that, the fabricated combo was covered by two plates (25 × 25 × 2.0 mm) on the top and bottom to form the PZT interface (Figure 5a). The material properties of the aluminum, the PZT 5 A [38], and superglue are listed in Table 4. The epoxy’s properties, which were used in the interface’s fabrication in this study, are also presented in this table.

Figure 5b shows three-quarters of a finite element (FE) model of the PZT interface, which was simulated using Comsol Multiphysics. The FE model had 968 elements, of which 100 were for the bonding layer, 100 for the PZT patch, 260 for the vibrating plate, 220 for the cover walls, and 288 for two cover plates. Quadratic hexahedron elements were used for the PZT interface. It was assumed that the PZT interface would be embedded in the concrete structures for impedance monitoring. Thus, all outer surfaces of the interface were assigned as fixed boundary conditions, and only the vibrating plate had the ability to oscillate in the structures.

**Figure 6:**
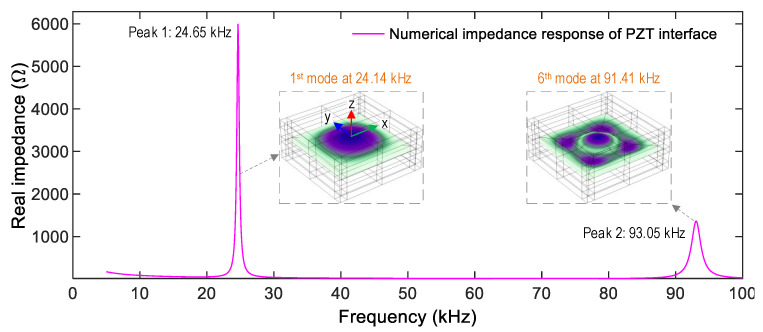
Numerical impedance responses of the PZT interface.

To acquire the impedance responses of the PZT interface, a 1 V harmonic excitation was assigned on the top surface of the PZT, while the bottom one was simulated as the ground electrode. Figure 6 shows the impedance signals of the PZT interface in the frequency range of 5–100 kHz with 1901 sweeping points. The PZT interface shows the first and second resonant impedance peaks at 24.65 kHz (Peak 1) and 93.05 kHz (Peak 2). Since the vibrating plate (flexible section) of the PZT interface had a square shape, the transverse displacements along the x-axis were identical to the ones along the y-axis. The impedance responses of Peaks 1–2 were the 1st (at 24.14 kHz) and 6th (at 91.41 kHz) flexural motions of the vibrating plate in the PZT interface (see Figure 6). Note that the low-frequency range of the PZT interface’s impedance signals can be pre-determined via the modal analysis, which is similar to previous studies [28,38].

### 2.3. Software Design for Raspberry Pi Platform Impedance Monitoring System

#### 2.3.1. Prototype of SSeL-Pi Impedance Monitoring System

**Figure 7:**
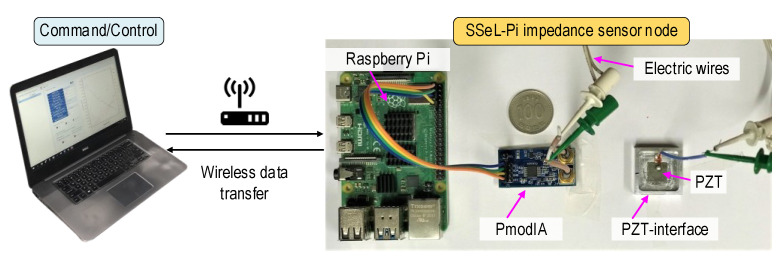
Prototype of impedance monitoring system ‘SSeL-Pi’.

A prototype of the SSeL-Pi impedance monitoring system was designed as shown in Figure 7. A prototype of the SSeL-Pi system was fabricated by a laptop, a Linux-operating Raspberry Pi platform, an impedance sensor board (PmodIA module), and a PZT interface device ensuring frequency responses less than 100 kHz. The way to connect the Raspberry Pi, PmodIA, and PZT interface is presented in Figure 3. The laptop controls the Raspberry Pi platform to command the PmodIA module for sensing impedance signals of the PZT sensor. The measurement command is transferred to the sensor node via WiFi communication. 

At the sensor node, Raspberry Pi automatically decodes the command to control the impedance sensor board. The PmodIA module introduces an excitation of 2 V into the PZT sensor at the designed frequency. At the same time, the corresponding impedance response at the exciting frequency is received backward from the sensor. The PZT interface device ensures the impedance signals (less than 100 kHz) that are sensitive to local damage and stress change. The decoding procedure of the input/output signals is carried out by the PmodIA module. Then, the impedance signatures are stored in the Raspberry Pi platform, and the saved data are wirelessly transferred to the laptop, where the post-process is conducted for the measured impedance features.

#### 2.3.2. Operation Scheme for Impedance Sensor Node

An operation scheme was designed for the Raspberry Pi platform impedance monitoring system. The impedance sensor node is controlled by an embedded Flask server, which is an application for building a web-server using Python language. The Flask server is used for REST API (Representational State Transfer Application Programming Interface) to communicate with other units. Since REST API uses the standard HTTP protocol, a unit to control the impedance sensor node can be easily developed by any programming language.

**Figure 8:**
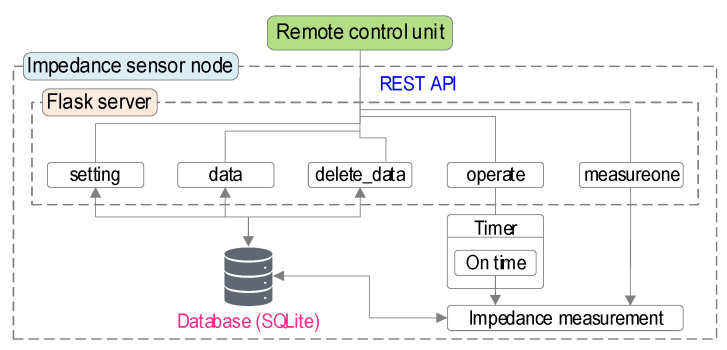
Control scheme of impedance monitoring system ‘SSeL-Pi’.

As shown in Figure 8, five routines were designed for the control scheme of the impedance sensor node, which includes “setting”, “operate”, “data”, “delete_data”, and “measureone”. The “setting” routine is used for setting impedance measurement parameters such as start frequency, frequency increment, and measuring interval or for getting stored parameters from the sensor node. The “operate” routine starts or stops scheduled monitoring by the pre-defined measuring interval. The “data” routine sends the measured data to the control unit. The “delete_data” routine deletes all measured data in the sensor node. The “measureone” routine measures impedance signals at once and sends the measured data. The setting parameters and measured data are stored in a database by SQLite DBMS (database management system). The sensor node returns JSON (JavaScript Object Notation) formatted data to the server.

#### 2.3.3. GUI Software for Impedance Monitoring

**Figure 9:**
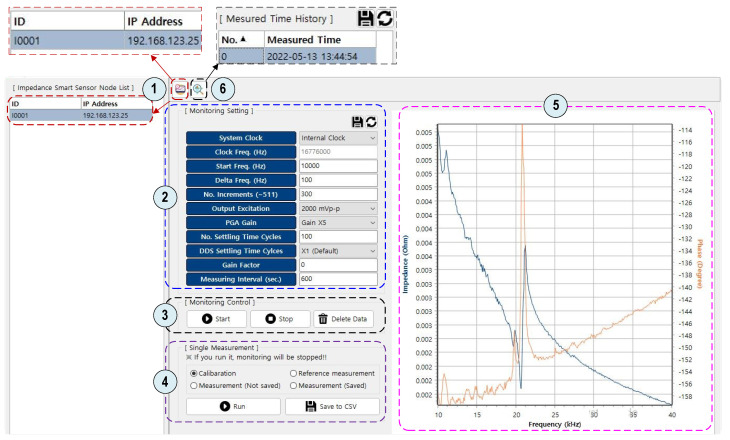
GUI software for Raspberry Pi system—’SSeL’ impedance sensor node.

As a general-purpose computer language, Delphi [39] was used to program the operating software of the SSeL-Pi impedance monitoring system. It is known that Delphi uses the Object Pascal (OP) programming language and provides an integrated development environment (IDE) for rapid application development of console software. The Delphi-based programming environment is free to academic users. A graphic user interface (GUI) software for the Raspberry Pi platform impedance monitoring was designed, as illustrated in Figure 9. As marked in the figure, the GUI software includes six main features to visualize the software interface, input command, and output results:

① Check and link to a ‘SSeL’ impedance sensor node. In this feature, the identity (ID) of the node is displayed. A previously set ID is a reference factor to distinguish a node from others. The tab “IP address” is also shown to visualize the dynamic IP of the node. A node’s ID must be input to identify the selected node for post-usage.
② Set impedance measurement parameters for the selected node. Eleven parameters and corresponding options should be selected according to the test requirement. The parameter ‘Measuring Interval’ should be set for periodic monitoring tasks.
③ Control the selected node to acquire impedance signatures in periodic monitoring. The measurement interval should be set correctly to measure impedance signals.
④ Test the status of the impedance measurement of the selected node. There are four options: Calibration, Measurement (No Save), Measurement (Save), and Reference measurement. These options should be selected based on the user’s purpose.
⑤ Display the real and phase parts of the impedance measurement results.
⑥ Save the measurement results into the master device (e.g., laptop).

## 3. Calibration of Impedance Monitoring System ‘SSeL-Pi’

The Raspberry Pi platform impedance sensing system (i.e., the prototype shown in Figure 7) was calibrated for accurate and stable performance. The calibration was carried out in the following four steps: (1) Acquisition of real and imaginary impedance, (2) Calculation of magnitude, gain factor, and phase angles, (3) Calibration of real and phase impedance, and (4) Extraction of correct real, and imaginary, and phase impedance. The calibration of impedance signals was conducted mainly on the impedance sensor board (Pmod IA).

**Figure 10:**
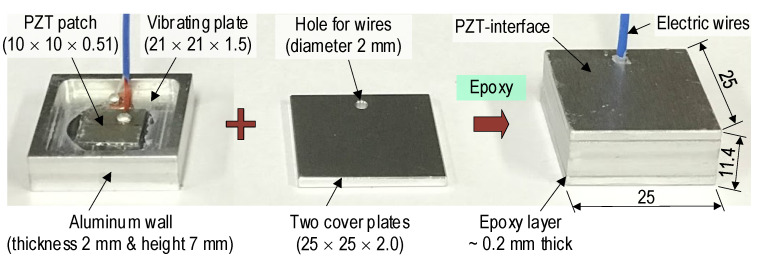
PZT interface device tested for calibration of impedance monitoring system ‘SSeL-Pi’. (dimension in mm).

Figure 10 shows the PZT interface device fabricated based on the conceptual design illustrated in Figure 4. A PZT 5 A patch [40] was surface-bonded on an aluminum vibrating plate (21 mm × 21 mm × 1.5 mm). The vibrating plate was supported by outer edges, which are aluminum walls (2 mm thickness and 7 mm height). Then, the fabricated combo was covered by two aluminum plates (25 × 25 × 2.0 mm). The cover plates had holes (diameter 2.0 mm) for passing electric wires.

**Figure 11:**
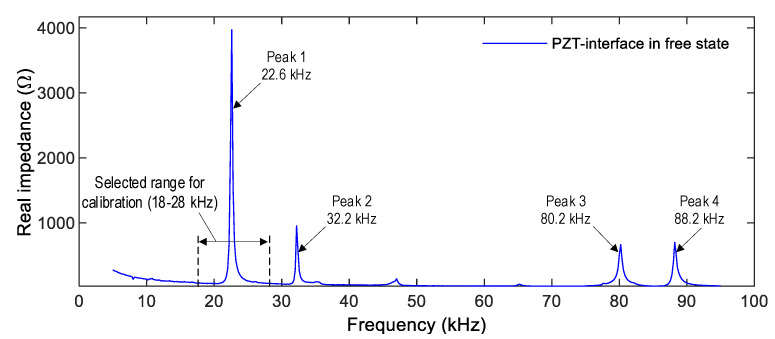
Impedance signal of PZT interface device measured by HIOKI-3532.

A series of calibration tests were performed on the PZT interface connected to the PmodIA board (see Figure 7). The PZT interface was freely placed on a table without any external loading. As the reference signature, the impedance signal of the PZT interface device was measured under the non-constraint boundary condition by using the HIOKI 3532 system. As shown in Figure 11, four impedance peaks (Peak 1 at 22.6 kHz, Peak 2 at 32.2 kHz, Peak 3 at 80.2 kHz, and Peak 4 at 88.2 kHz) were acquired in the frequency range of 5–95 kHz. As compared to the numerical impedance signal (see Figure 6), the experimental impedance signature of the PZT interface sample had a similar pattern at Peak 1 (highest peak). Furthermore, there was a difference between experimental and numerical impedance signals in other peaks. The difference could be induced by the boundary conditions of the PZT interface (non-constraint in the experimental measurement and fixed in the numerical analysis) and the effect of the fabrication process. It is known that the resonant impedance frequency bands represent meaningful structural characteristics [38,41]. The frequency range of 18–28 kHz (containing the highest peak—Peak 1) was selected for the calibration test. The calibration procedure was demonstrated by comparing it with the HIOKI 3532 analyzer.

Step 1: Acquisition of Real ($R_{P}$) and Imaginary ($I_{P}$) Impedance

**Figure 12:**
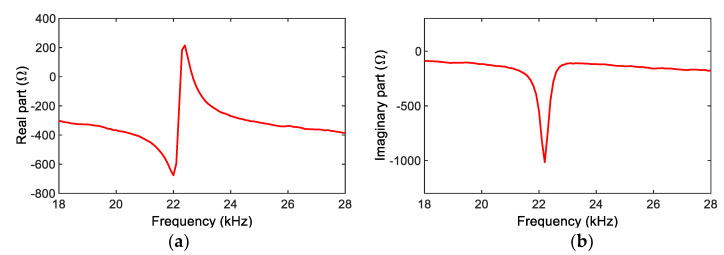
Impedance responses of PZT interface measured by SSeL-Pi system. (**a**) Real part $R_{P}$, (**b**) Imaginary part $I_{P}$.

**Figure 13:**
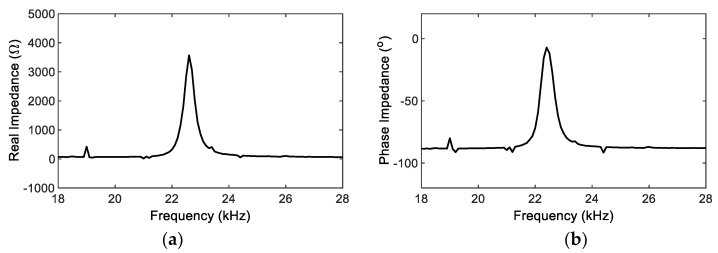
Impedance responses of PZT interface measured by HIOKI-3532. (**a**) Real impedance $R_{H}$, (**b**) Phase impedance $\varphi_{H}$.

An impedance board (PmodIA) was connected to the PZT sensor interface, and a wide range of frequency-domain excitation was applied to the PZT sensor. Figure 12 shows real ($R_{P}$) and imaginary ($I_{P}$) parts of impedance responses measured by the SSeL-Pi system. Figure 13 shows the corresponding impedance signature ($R_{H}$) and phase ($\varphi_{H}$) measured by the HIOKI 3532 system. The frequency band of 18~28 kHz was selected for both the SSeL-Pi and HIOKI systems.

Step 2: Calculation of Impedance Magnitude $M_{P}$, Gain Factor $G_{1}$, and Phase $\varphi_{P}$, $\varphi_{1}$_._

For the real and imaginary impedance data of the SSeL-Pi system (see Figure 12), the impedance magnitude was calculated as follows:

(3) $$M_{P}=\sqrt{R_{P}^{2}+I_{P}^{2}}$$

The gain factor $G_{1}$ was obtained for the calculated magnitude $M_{P}$ by treating the real impedance data $R_{H}$ of the HIOKI 3532 (see Figure 13a) as the reference:

(4) $$G_{1}=\frac{1}{M_{P}\times R_{H}}$$

At each sensor point of the SSeL-Pi system, the phase angle $\varphi_{P}$ was calculated from the real and imaginary data, $R_{P}$ and $I_{P}$:

(5) $$\varphi_{P}=\tan^{-1}\left(\frac{I_{P}}{R_{P}}\right)\times \frac{180^{o}}{\pi }$$

in which the signs of $R_{P}$ and $I_{P}$ components were positive. In the case of the positive $R_{P}$ and negative $I_{P}$, the phase angle should add 180° into Equation (5) [16]. For the calibration of the impedance sensing system, the gap of phase angles between $\varphi_{P}$ (SSeL-Pi system) and $\varphi_{H}$ (HIOKI-3532) was calculated as $\varphi_{1}$:

(6) $$\varphi_{1}=\varphi_{P}-\varphi_{H}$$

**Figure 14:**
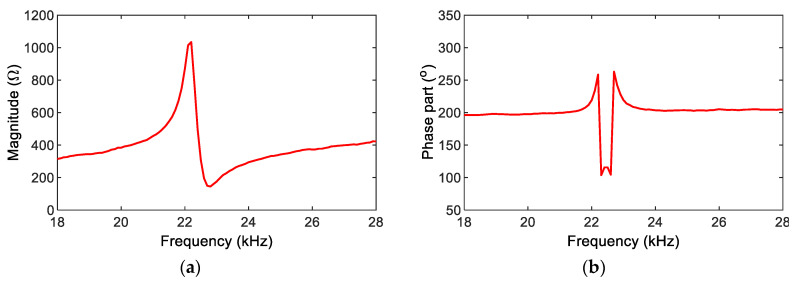
Calculation of impedance magnitude and phase angle for Raspberry Pi system. (**a**) Magnitude $M_{P}$, (**b**) Phase angle $\varphi_{P}$.

**Figure 15:**
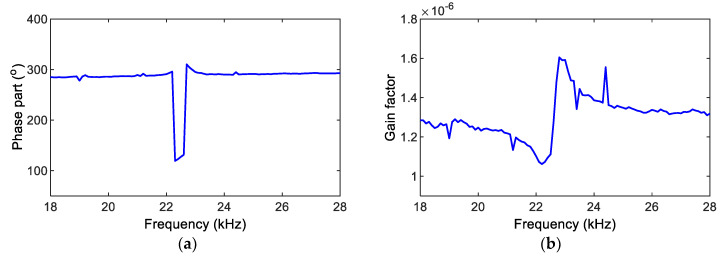
Calculation of impedance phase gap and gain factor for SSeL-Pi system. (**a**) Phase gap $\varphi_{1}$, (**b**) Gain factor $G_{1}$.

The impedance magnitude $M_{P}$ and phase angle $\varphi_{P}$ were calculated for the SSeL-Pi system, as shown in Figure 14. The impedance phase gap and gain factor were calculated for the SSeL-Pi system, as also shown in Figure 15.

Step 3: Calibration of Real and Phase Impedance

As previously observed in Figure 15, the impedance phase gap $\varphi_{1}$ and gain factor $G_{1}$ were different along the monitored frequency (f). Linear regression models were established to manage these variations. The phase angle and gain factor values were standardized on the basis of the phase angle $\left(\varphi_{1-i},f_{i}\right)$ obtained in the *ith* frequency and the gain factor $\left(G_{1-i},f_{i}\right)$ obtained in the *ith* frequency (as described in Step 2). 

**Figure 16:**
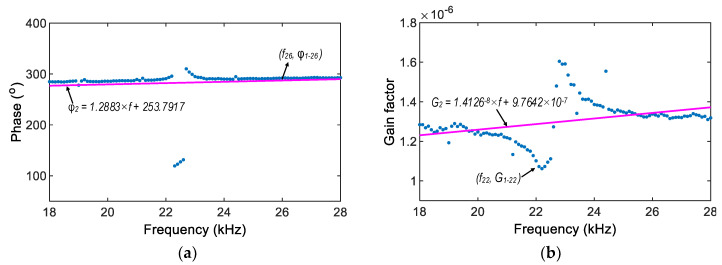
Regression models for impedance phase angle and gain factor. (**a**) Phase fit $\varphi_{2}$, (**b**) Gain factor fit $G_{2}$.

Figure 16 shows two regression models for estimating the phase fit $\varphi_{2}$ (see Figure 16a), and the gain factor fit $G_{2}$ (see Figure 16b) at investigated frequencies. Then, the impedance magnitude and phase angle were corrected as follows:

(7) $$Z=\frac{1}{G_{2}\times M_{P}}$$

(8) $$\theta =\varphi_{P}-\varphi_{2}$$

in which Z and θ denote the corrected impedance magnitude and corrected phase angle.

Step 4: Extraction of Correct Real, Imaginary, and Phase Impedance

The real $\left(R_{C}\right)$, imaginary $\left(I_{C}\right)$, and phase ($\left(\varphi_{C}\right)$ components of the impedance were calibrated based on the corrected magnitude Z and the corrected phase angle θ described in Step 3. The calibration was made by the vector projection of the impedance magnitude and phase angle onto the real and imaginary impedance components, as follows:

(9) $$\left|R_{C}\right|=\left|Z\right|\times \cos(\theta )$$

(10) $$\left|I_{C}\right|=\left|Z\right|\times \sin(\theta )$$

(11) $$\varphi_{C}=-\theta$$

**Figure 17:**
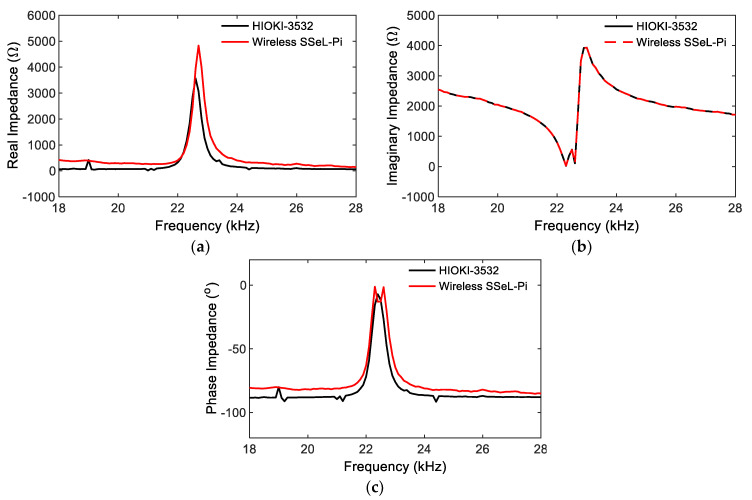
Performance of impedance calibration between SSeL-Pi and HIOKI-3532. (**a**) Real impedance $R_{C}$, (**b**) Imaginary impedance $I_{C}$, (**c**) Phase impedance $\varphi_{C}$.

The accuracy of the calibrated impedance signals of the SSeL-Pi system was evaluated by comparing it to the HIOKI 3532 system. As shown in Figure 17, the imaginary impedance signal measured by the SSeL-Pi impedance sensing node had a good consistency with the one measured by the HIOKI 3532 system. The real impedance and phase impedance components were relatively similar, and the frequencies corresponding to the peaks were almost the same. Accordingly, the performance of the SSeL-Pi system could be successfully guaranteed after the calibration. 

## 4. Experimental Evaluation of Impedance Monitoring System ‘SSeL-Pi’

### 4.1. Testing Setup and Test Scenarios

**Figure 18:**
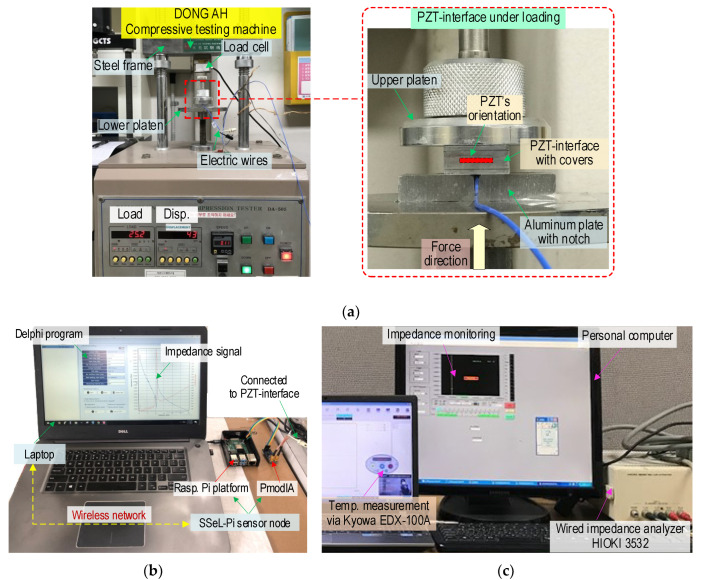
Impedance measurement of PZT interface device via SSeL-Pi and HIOKI-3532. (**a**) PZT interface device under compression test, (**b**) SSeL-Pi system, (**c**) HIOKI 3532 system.

As shown in Figure 18, laboratory experiments were performed on PZT interfaces under compressive loadings to acquire impedance signals from the SSeL-Pi system. A PZT sensor interface (see Figure 10) was positioned in the compression testing machine, as shown in Figure 18a. Compression forces controlled by a load cell were applied to the PZT interface with a loading speed of 0.05 mm/min. Two PZT interfaces (i.e., two test samples), PZT 1 and PZT 2 were measured for impedance responses under the z-directional compressive loading. As the loading scenarios, the forces were incrementally applied from P1 (2.0 kN) to P5 (4.0 kN) with an interval of 0.5 kN. Note that P1 (2.0 kN) was treated as the baseline intact state to compare with P2~P5 loading cases.

As shown in Figure 18b, the SSeL-Pi system was utilized to measure impedance responses of the two PZT interfaces (PZT 1 and PZT 2) as the compressive loadings varied from P1 to P5. For the z-directional loadings, the impedance signals were measured at 31 points in a frequency range of 20.5–23.5 kHz by a constant interval of 0.1 kHz. At the same time, the impedance analyzer HIOKI 3532 (see Figure 18c) was set up to measure the impedance signals of the two PZT interfaces (PZT 1 and PZT 2) for the same loading scenarios. The impedance signatures measured from the HIOKI 3532 system were used as the reference to evaluate the confidence levels of impedance signatures measured from the SSeL-Pi sensor node. The laboratory temperature was kept near constant, around 20 °C (measured via Kyowa EDX-100A), to minimize the effect of temperature variation on impedance features. For each loading case, four ensembles of impedance responses were measured to determine an upper control limit (UCL) threshold. 

### 4.2. Impedance Measurement of Raspberry Pi Impedance Monitoring System

#### 4.2.1. Impedance Responses of PZT Interface under Compression

**Figure 19:**
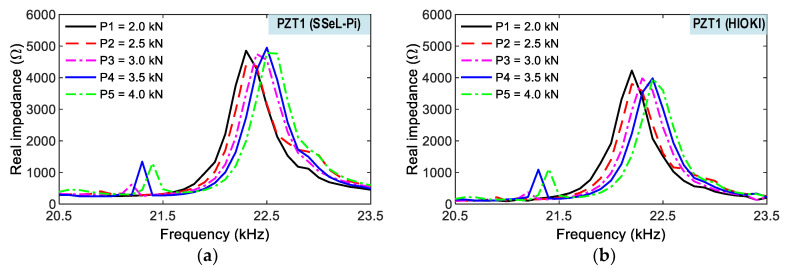
Impedance signals of PZT 1 for compressive loadings P1–P5. (**a**) SSeL-Pi, (**b**) HIOKI-3532.

**Figure 20:**
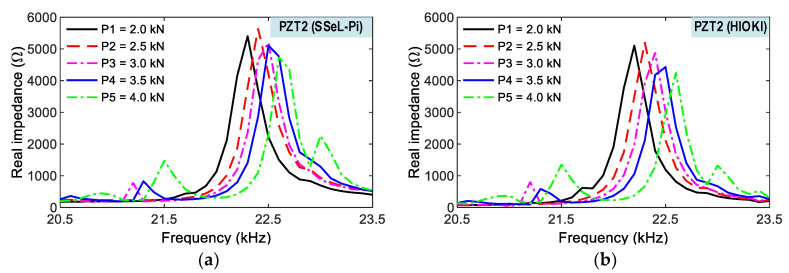
Impedance signals of PZT 2 for compressive loadings P1–P5. (**a**) SSeL-Pi, (**b**) HIOKI-3532.

As shown in Figure 19, impedance responses of a PZT interface (PZT 1) were measured via the wireless SSeL-Pi and wired HIOKI-3532, respectively. For the five loading cases (i.e., P1–P5), the impedance signals of the two systems had similar patterns for the series of loadings. The impedance signals of both systems shifted to the right with the increment of compressive loadings. The impedance signals measured from both systems consistently responded to the series of loadings. Additionally, Figure 20 shows the impedance responses of the other interface (PZT 2) under five loading cases (i.e., P1–P5). Like the PZT 1, the impedance signals measured from the wireless SSeL-Pi and wired HIOKI 3532 were similar in their patterns. The variation tendency of the experimental impedance responses from the SSeL-Pi system was consistent with those from the HIOKI 3532 system. As observed in Figure 19 and Figure 20, the alteration in impedance responses of PZT 2 was higher than those of PZT 1. The difference could be caused by the sensor fabrication, bonding condition, and compressive testing setup.

#### 4.2.2. Frequency Shifts in Impedance Responses of PZT Interfaces under Compression

**Figure 21:**
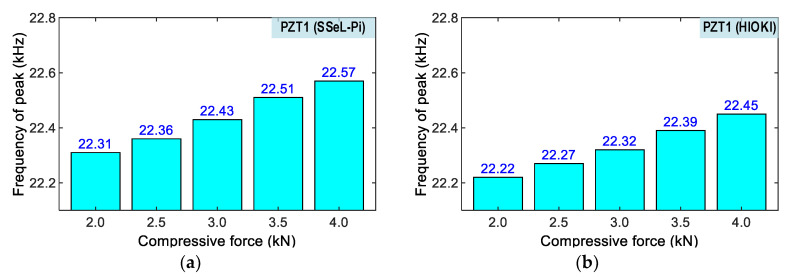
Frequency shifts of PZT 1 corresponding to compressive loadings P1–P5. (**a**) SSeL-Pi, (**b**) HIOKI-3532.

**Figure 22:**
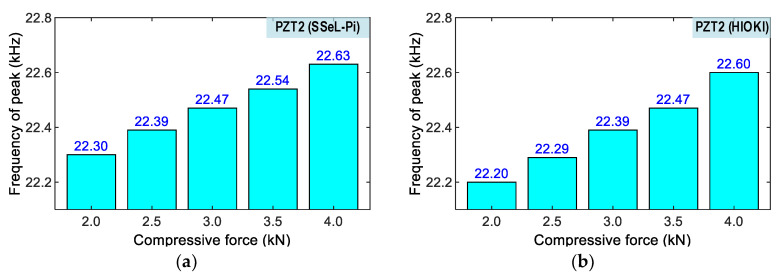
Frequency shifts of PZT 2 corresponding to compressive loadings P1–P5. (**a**) SSeL-Pi, (**b**) HIOKI-3532.

Figure 21 and Figure 22 show peak impedance frequencies of the two PZT interfaces (PZT 1 and PZT 2) that were acquired by the SSeL-Pi and HIOKI 3532 systems under applied forces P1–P5. There were slight differences in the peak frequencies of PZTs 1–2 measured by the SSeL-Pi and HIOKI 3532 under the reference case P1. By comparing to the reference P1, the peak frequencies increased linearly under the incremental loadings P2–P5. For PZT 1 (see Figure 21), the peaks rose from 0.05 kHz (0.25%) to 0.26 kHz (1.18%), which was measured via the SSeL-Pi, and from 0.05 kHz (0.25%) to 0.23 kHz (1.06%), which was measured via the HIOKI 3532. For PZT 2 (see Figure 22), the peaks increased from 0.09 kHz (0.40%) to 0.33 kHz (1.48%), which was measured via the SSeL-Pi, and from 0.09 kHz (0.42%) to 0.40 kHz (1.79%), which was measured via the HIOKI 3532.

As analyzed, it can be observed that there was a similarity in the frequency shifts in the impedance responses measured by the SSeL-Pi and HIOKI 3532 for both PZTs 1–2, thus suggesting the potential of the SSeL-Pi system for impedance measurement. Furthermore, the linear variations of the peak frequencies in the impedance signals of the PZT interfaces (PZTs 1–2) showed the feasibility of the new PZT interface device for impedance monitoring.

#### 4.2.3. RMSD Changes in Impedance Responses of PZT Interfaces under Compression

To quantify the changes in impedance signals, the RMSD (root mean square deviation) index is commonly used as a damage indicator for the characterization of structural damage.

The RMSD index is computed as follows [5]:

(12) $$RMSD\left(Z,Z^{*}\right)=\sqrt{\left(\sum_{i=1}^{N}{\left[Z^{*}(\omega_{i})-Z(\omega_{i})\right]}^{2}\right)/\sum_{i=1}^{N}{\left[Z(\omega_{i})\right]}^{2}}$$

where $Z(\omega_{i})$ and $Z^{*}(\omega_{i})$ are the impedance signals in the intact and damaged states of structure at frequency, respectively, and N denotes the number of frequency sampling points in the sweep.

**Figure 23:**
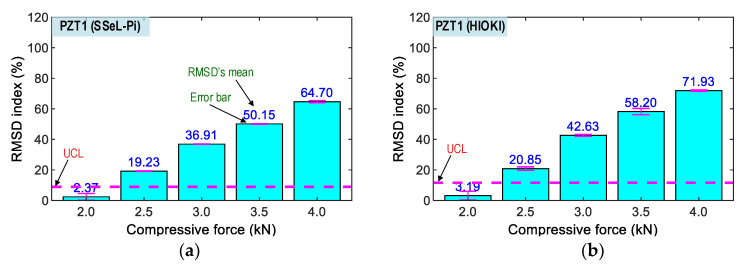
Impedance features of PZT 1 corresponding to compressive loadings P1–P5. (**a**) SSeL-Pi, (**b**) HIOKI-3532.

**Figure 24:**
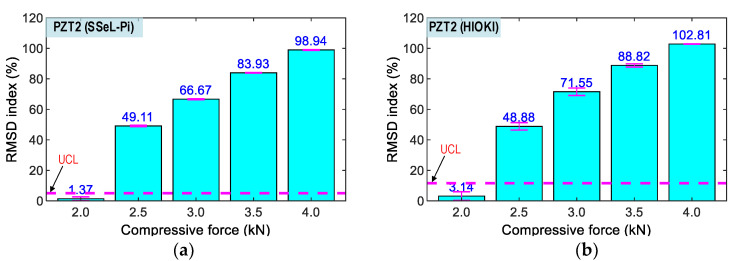
Impedance features of PZT 2 corresponding to compressive loadings P1–P5. (**a**) SSeL-Pi, (**b**) HIOKI-3532.

Figure 23 and Figure 24, respectively, show the RMSD indices of the impedance signatures of PZT 1 and PZT 2 computed for the five compressive loadings P1–P5. The upper control limit UCL [42] was calculated using the impedance signals at the applied force P1 (as the reference). The error bar was also computed for each loading case. Generally, an upward trend in the percentage of RMSD indices was found as the compressive force rose. The RMSD indices were negligible in the intact case (P1), but they were linearly increased and beyond the UCLs in other cases (P2–P5), thus suggesting that the variations of the compressive forces were successfully monitored. The small error bars in Figure 23 and Figure 24 also pointed out that the impedance signals were relatively stable. Note that a similar tendency was observed in the SSeL-Pi and HIOKI-3532. Thus, the analyzed results demonstrated the feasibility of the SSeL-Pi system for impedance monitoring in the PZT interfaces.

#### 4.2.4. Empirical Relationship of Impedance Features and Stress

**Figure 25:**
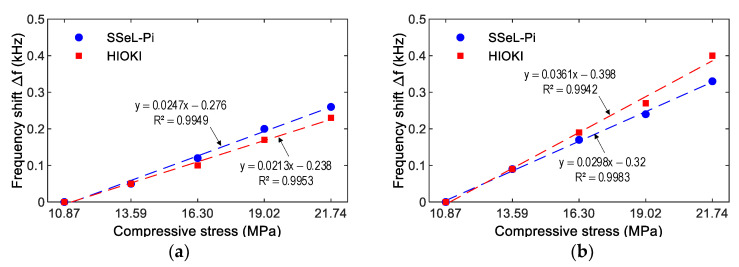
Relationship between frequency shift and corresponding compressive stress. (**a**) PZT1, (**b**) PZT2.

**Figure 26:**
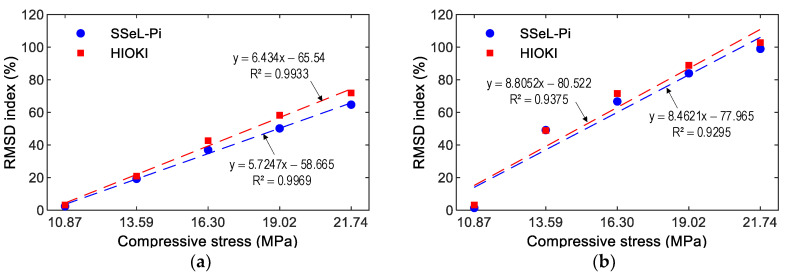
Relationship between RMSD index and corresponding compressive stress. (**a**) PZT1, (**b**) PZT2.

Figure 25 and Figure 26 show the relationships between the impedance features of PZTs 1–2 and compressive stresses induced by loading cases P1–P5. The relationships were built based on impedance signals, which were obtained from the SSeL-Pi and HIOKI 3532 systems. These correlations were compared to each other to show the potential of the proposed measuring system for impedance measurement. 

Figure 25 presents the correlation between the frequency shift ∆f and the stress. As observed in this figure, strong positive linear relations could be found between the frequency shifts and stresses. The correlation coefficients $R^{2}$ were beyond 0.994 for both PZTs 1–2. The same tendency could be observed for the correlation between the RMSD index and the stress, as shown in Figure 26. It can be noted that significant correlations could be found between the RMSD indices and stresses. $R^{2}$ was beyond 0.993 for PZT 1 and 0.929 for PZT 2. Note that the strong relationships between the impedance features of PZT interfaces (i.e., PZTs 1–2) could be observed. Furthermore, the relationships built on impedance signals of the SSeL-Pi and HIOKI 3532 (see Figure 25 and Figure 26) were quite consistent. Therefore, it can be concluded that the new wireless impedance sensing node SSeL-Pi has a high potential for impedance-based structural health monitoring.

From the experimental analyses, the following observations were made to verify the feasibility of the Raspberry Pi platform impedance monitoring system “SSeL-Pi”: (1) the measurable impedance frequency range of less than 100 kHz was controlled using the new PZT interface device; (2) the impedance analyses using signals obtained from the SSeL-Pi were quite consistent with the ones using signatures measured from the commercial analyzer HIOKI 3532; (3) the impedance features measured by the PZT interfaces were relatively similar and sensitive to the variation in compressive forces; and (4) the relationship between impedance signatures and stresses could be used to quantitatively monitor stress change in structures using the new PZT interface.

It is also noted that more tests on the SSeL-Pi system should be conducted in the future to verify the practicability of the developed system and also to determine empirical formulas on impedance signatures and compressive forces, which would be reliable enough for impedance-based stress monitoring in concrete structures.

## 5. Concluding Remarks

The wireless impedance monitoring system was developed to have cheap, mobile, and handy practical features as compared to wired commercial impedance analyzers (e.g., HIOKI 3532 or HP4149A). The Raspberry Pi platform sensor node was designed to acquire impedance signals by collaborating with the PZT interface technique. The software scheme was designed to operate the Raspberry Pi platform and impedance sensor node. The calibration procedure was designed for the impedance sensor node. The feasibility of the proposed Raspberry Pi platform SSeL-Pi system was experimentally evaluated for PZT interfaces that were subjected to various compressive loadings. 

From the experimental evaluation, at least four concluding remarks can be made as follows: Firstly, the prototype of the ‘SSeL-Pi’ impedance monitoring system was successfully fabricated by the remote-control laptop, Linux-operating Raspberry Pi platform, impedance sensor board (PmodIA module), and the PZT interface device ensuring frequency responses of less than 100 kHz. Secondly, the Delphi-based GUI software was programmed to visualize the input/output parameters for the Raspberry Pi platform and automate the signal acquisition procedure for the PmodIA module via the PZT interface. Thirdly, the calibration of the impedance signals was carried out on the PmodIA module, from the acquisition of real and imaginary impedance to the adjustment of them, with respect to a commercial impedance analyzer, HIOKI-3532. Finally, the impedance responses of the PZT interface that was subjected to a series of compressive loadings were accurately measured by the SSeL-Pi system compared to the HIOKI-3532. There were strong linear relationships between impedance features (frequency shift and RMSD index) and compressive stresses of the PZT interface, thus suggesting that the developed SSeL-Pi system has the potential for stress variation monitoring in concrete structures. 

## Figures and Tables

**Table 1:** Raspberry Pi platform impedance monitoring system ‘SSeL-Pi’ versus commercial impedance analyzer ‘HIOKI-3532’.

Parameter	SSeL-Pi System [22,27]	HIOKI 3532 [30]
Impedance range	100 Ω–10 MΩ	10 mΩ–200 MΩ
Frequency range	1 kHz–100 kHz	42 Hz–5 mHz
Voltage peak-to-peak	2 V_p-p_	14 V_p-p_
Cost	US$280	US$15,000
Dimension	88 × 58 × 19.5 mm	352 × 323 × 124 mm
Weight	80 g	6.5 kg

**Table 2:** Main specifications of Raspberry Pi 4 B.

No.	Name	Feature	No.	Name	Feature
1	mCPU	64-bit quad-core Cortex-A72 (1.5 GHz)	5	GPIO	40 pinouts
2	RAM	4 GB LPDDR4	6	Storage	MicroSD-16 GB
3	Wireless	802.11 ac (2.4/5 GHz)	7	USB ports	2 × USB 3.0, 2 × USB 2.0
4	Power supply	USB Type-C/5 V and 3 A	8	HDMI ports	2 × Micro-HDMI up to 1080 p60

**Table 3:** Initial setup of impedance sensing module ‘PmodIA’.

Specification	Value	Unit
Internal oscillator frequency	16.776	mHz
Current-to-voltage amplifier gain resistor	20,000	Ohm
Programmable gain amplifier (PGA gain)	×5	-
Output excitation voltage	2 V_p-p_	V
Number of settling time cycles	100	-
Supply voltage	3.3	V

**Table 4:** Material properties of aluminum, PZT patch, and bonding layers.

Properties	Aluminum 6061-T6	PZT 5 A	Bonding Layer Super Glue	Bonding Layer Epoxy
Young’s modulus, E (GPa)	68.9	62.1	5	0.75
Poisson’s ratio, ν	0.33	0.35	0.38	0.3
Mass density, ρ (kg/m^3^)	2700	7750	1700	1090
Damping loss factor, η	0.02	0.0125		0.02
Yield strength, $\sigma_{y}$ (MPa)	241			
Compressive strength, $\sigma_{c}$ (MPa)				32.3
Dielectric constant, $\varepsilon_{33}^{T}$ (F/m)		1.53 × 10^−8^		
Coupling constant, $d_{31}$ (m/V)		−1.71 × 10^−10^		
Dielectric loss factor, δ		0.015		

## Data Availability

Data available on reasonable request from the corresponding author.
